# The Clinical Society of Maryland

**Published:** 1892-02

**Authors:** W. T. Watson

**Affiliations:** Secretary; 1603 N. Broadway, Baltimore


### Society Reports.

### THE CLINICAL SOCIETY OF MARYLAND.

### 259TH REGULAR MEETING, BALTIMORE, DECEMBER 18, 1891.

The society was called to order by the President, DR. ROBERT JOHNSON.

DR. C. W. MITCHELL read a paper entitled,

AFTER INFLAMMATION—WHAT ?

DR. WM. B. CANFIELD read a paper on

DUST AS A CAUSATIVE FACTOR IN PULMONARY DISEASE.

The various kinds of dust may be divided into animal, mineral and vegetable. Opinions differ as to which kinds are most dangerous when inhaled. That which is generated in brush factories is animal and very harmful. Makers of hats, especially felt hats, suffer much from the dust evolved. The vegetable dust that does the greatest and most lasting injury to the lungs is that generated in tobacco factories. This dust has not only a mechanical action, but has also poisonous effects.

It is in connection with the inhalation of mineral in it that the greatest amount of scientific investigation has been made, especially in relation to the diseases called the consumption of grinders, miners, potters, etc. Anthracosis, silicosis, siderosis, chalicosis, tabacosis and other kindred names have been suggested to describe a similar condition produced by various kinds of dust. Zenker has handed down the word "pneumonoxoniosis" to cover all these conditions. The history of these cases is very much alike. They begin with simple bronchitis which gradually becomes chronic. They are usually non-tuberculous, at least at the beginning tuberculosis complication is only an accident.

Where one is exposed to an atmosphere of dust the contact of this dust with the sensitive nasal and laryngeal mucous membrane sets up coughing and sneezing, and much of the dust is expelled, and for a time no harm results; but a continued exposure to this dust causes a congestion of the mucous membrane of the nose and breathing passages and in time an inflammation of the whole tract; the ciliated epithelium loses its power and dust finds its way to the ultimate ends of the lung tubules. When the individual is asleep or absent from this irritation, the ciliated epithelium gets rid of a large part of this foreign substance, and the wandering cells may close around some of this dust and try to carry it off or render it harmless by burying it in a lymphatic gland. Much, however, finds its way either through the epithelium or between the cells into the submucous layer, getting into contact with the connective tissue of the alveoli, and by irritation causing a hypertrophy of this tissue, and a condition resembling chronic interstitial pneumonia or fibroid phthisis. The general opinion seems to be that the fibroid condition seems to oppose a direct barrier to the growth and multiplication of the bacillus tuberculosis, and in large tracts of lung tissue converted into this material often not a bacillus could be detected. In one of the author's cases bacilli were found in abundance, and yet two year afterward the man reported himself as entirely well.

The color of the expectoration is a prominent sign in these cases. In one case of the author, a stoker, the expectoration still continues absolutely black at times, and always tinged, although it is almost two years since he gave up his occupation. Examination of this sputum under the microscope showed it to contain in abundance carrier cells which in all cases contained pigment and in some instances the black crystalline coal could be recognized within these cells. This pigment and foreign material has a tendency to collect at the apices of the lungs, and is only present at the bases when the dust inhaled is excessive in amount and exposure prolonged.

In diagnosis, physical signs do not yield as much as the microscope. By the microscope we see the cells containing the dust. In the author's cases (four) râles were heard on auscultation, but nothing marked was obtained on percussion.

The prognosis is good if the man has not worked too long at the occupation.

The treatment is to take the patient from his dangerous occupation, when improvement begins at once. Owners of large factories are adopting stringent prophylactic measures in order that they may not lose so many good workmen. The best methods are: 1. To prevent the formation or escape of dust by using wet grinding or by grinding in closed vessels. This is not always practicable. 2. To prevent inhalation of dust by wearing respirators, etc. But these are uncomfortable, and the men remove them at every opportunity. 3. The removal of dust as fast as it is produced by using fans and air shafts. This is by far the best plan.

Still further the following rules should be enforced: 1. Workmen should change their outer clothing after work. 2. They should keep their faces and hands as clean as their work will allow. 3. They should never be allowed to eat in the workroom.

DR. RANDOLPH WINSLOW related

### A CASE OF ELEPHANTIASIS SCROTI.

J. C., colored, aged 44 years, was admitted to the University Hospital, September 7, 1891, on account of enlargement of the scrotum and perineum. His father died of meningitis, and his mother of phthisis. Patient is one of seven children, six of whom died of phthisis. He had measles in childhood, typhoid fever at twenty-one and gonorrhœa about eight years ago. The present disease began about three years ago, with slight thickening of the tissues of the scrotum, penis and perineum, the infiltration first showing itself in the skin of the scrotum and increasing slowly until at the time of his admission, the scrotum was enormously enlarged and reached one-third of the distance to the knee. There were a number of suppurating sinuses and superficial abscesses in the scrotum and perineum. There was some pain. The tissues of the scrotum were brawny and very little impression could be made on them by pressure. The perineum was composed of similar tissue and was enormously hypertrophied. The skin of the penis was also thickened, but retained its suppleness and the prepuce could easily be retracted. The patient said that his virile powers were unimpaired. He was a sailor, but had never been much beyond the coast of this country and had never resided in a tropical country. Several efforts to detect the filaria sanguinis hominis were unsuccessful.

The sinuses were incised, and a long incision made in the perineum to relieve tension and allow the lymph and blood vessels to empty themselves. He was placed upon iodide of potassium, as syphilis could not be excluded. He did not improve, and excision of the scrotum and perineal hypertrophy was performed October 1st. The skin and subcutaneous tissues were very dense and thick and freely supplied with blood vessels. The testicles were carefully dissected out and were uninjured. The gap in the perineum was closed with sutures, but there was not sufficient tissue to cover the testicles, hence lateral incisions were made in the contiguous skin and strips of skin dissected up and brought over so as to form a new scrotum. The tension was great and the stitches cut out allowing the flaps to separate considerably. Healing was effected under about five dressings, and he was discharged well on November 8, relieved of pain and discomfort, and ready again to resume his ordinary avocations.

W. T. WATSON, M. D.,
1603 *N. Broadway*, *Baltimore*. Secretary.

---

WE learn that Mr. William Rose, of London, has now operated four times for tic douloureux by removing the Gasserian ganglion. Patients are said to have been benefited.

---

THE next meeting of the Southern Surgical and Gynecological Association will be held in Louisville, Ky., November next. President, Dr. J. McFadden Gaston, Atlanta, Ga.

---

IT is stated in the secular press that the bacillus of influenza has at last been discovered by Dr. Pfeiffer, the son-in-law of Dr. Koch. The bacillus is said to be the smallest yet observed.

